# Use of a two-handed model to improve comprehension of ventricular outflow tract anatomy

**DOI:** 10.1186/s12909-023-04083-w

**Published:** 2023-02-08

**Authors:** Xiang Xue, Xianyuan Luo, Zhaoyang Liu, Yun Jin

**Affiliations:** Division of Cardiology, Changzhou Geriatric Hospital Affiliated With Soochow University (Changzhou No.7 People’s Hospital), 288 Yanling East Road, Changzhou 213011 Jiangsu, China

**Keywords:** Cardiac anatomy, Cardiac electrophysiology, Heart model, Ventricular outflow tract, Anatomy teaching

## Abstract

**Background:**

Mastering cardiac anatomy is a formidable obstacle in the learning process for cardiac electrophysiology trainees. The complex three-dimensional characteristics and contiguous relationship of the ventricular outflow tract are particularly difficult to visualize with the limited study methods available. The hands can recreate a morphology similar to the ventricular outflow tract; this study explored whether a two-handed model of the heart helps electrophysiology trainees improve their understanding of ventricular outflow tract anatomy.

**Methods:**

After an initial assessment, trainees were randomly placed into variable and control groups. Subsequently, all trainees learned the outflow tract anatomy using routine methods, with the variable group receiving additional instruction using the two-handed model. One day and one week after the course conclusion, knowledge of the ventricular outflow tract anatomy was assessed for the participants in both groups.

**Results:**

Thirty-eight trainees participated (19 in each group). The median scores obtained for the first, second, and third tests were 38 (24,55), 80 (70,86), and 75 (70,81) points, respectively. In the second test, trainees in the variable group had a mean score 6.8 points higher than those in the control group (*p* = 0.103); in the last test, the mean score was 9.7 points higher in the variable group than in the control group (*p* = 0.003).

**Conclusions:**

It is convenient to use hands to create a model representing the ventricular outflow tract. Trainees using this model had a better understanding and retention of the ventricular outflow tract anatomy compared to those of the control group.

**Supplementary Information:**

The online version contains supplementary material available at 10.1186/s12909-023-04083-w.

## Background

Cardiac anatomy is one of the most challenging subjects to learn because of its complex, multicavity, and three-dimensional morphology [1]. The cardiac outflow tract is particularly difficult to understand due to its complex developmental anatomy and the nonintuitive anatomical relationships among the various structures [2]. The ventricular outflow tract is commonly implicated as the site of origin of various arrhythmias [3–5]. A thorough understanding of the ventricular outflow tract anatomy will facilitate better localization of the origin of ventricular arrhythmias and improve the efficacy of catheter ablation [6]. Furthermore, the ventricular outflow tract is adjacent to many important anatomical structures, such as the left main coronary artery (LMCA), the bundle of His, and the atrial appendages [7].

Knowledge of cardiac anatomy can be gained through the following traditional methods: anatomy textbooks, heart models, pig hearts, cadaveric dissections and anatomic atlases. In the past 20 years, technology-enhanced learning (TEL) resources have had a tremendous impact on medical anatomy education, such as three-dimensional (3D) visualization [8], virtual reality [9], virtual dissection of computed tomography datasets [10], social media [11], 3D printing of replica specimens [12], and open online courses [13]. These methods can often be combined for better learning and visualization. Although anatomy textbooks describe structures in detail, words and pictorial representations are often difficult for beginners to grasp. In contrast, the use of TEL is more intuitive. A comprehensive review of the assessment of TEL has confirmed the satisfaction and enjoyment of students [14]. However, many scholars have questioned whether TEL can provide adequate learning gains [15, 16]. As the underlying costs of introducing a TEL resource into a course are relatively high, cost–feasibility analysis discourages the use of TEL [17]. Art-based activities, including body painting and materials-based modeling, are another effective way to learn anatomy [18]. Although they are cost-effective and interesting, Chytas et al. reported doubts about the impact of body painting because examination scores of those who did and did not use the method did not differ significantly [19]. Regardless of the method employed, the first principle of anatomical learning is to describe the structures as viewed in the so-called anatomical position [20]. Following this principle, the anatomically appropriate position of the heart and its components can be visualized in relation to the rest of the body. The main disadvantage of the abovementioned methods is that they may not always be readily available; therefore, they fail to provide real-time guidance during an operation.

Despite the many teaching methods available to them, electrophysiological trainees still find it challenging to learn cardiac anatomy in the beginning, especially that of the outflow tract. Therefore, educators must implement the most suitable learning theory to help beginners in electrophysiology improve their understanding of outflow tract anatomy. Inspired by the training of physician assistants, constructivism is an appropriate theory [21]. John Dewey, Lev Vygotsky, and Jean Piaget are the three representatives of this theory [22], which includes the following principles [23, 24]. Learning is an active process, and acquiring knowledge is fundamentally a social construct. Everyone's perspectives are valued, and all adhere to a mental model of learning. The clinical teaching of electrophysiology students includes three key elements: coaching, modeling, and scaffolding. "Modeling" means making full use of case-based examples. "Scaffolding" means temporary support is given by guidance on difficult tasks [25]. The two most significant differences between the teaching of electrophysiological centers and schools are as follows: 1. In centers, the most important learning examples are patients undergoing radiofrequency ablation, and 2. educators at the hospital tend to speak less because they have to focus more on patients. Hence, students expect a virtual teacher who will accompany them throughout the learning process as a facilitator, especially during operations, and they feel a sense of ease in asking the "teacher" any questions.

When playing a hand shadow game with children, we found the bimanual morphology strikingly similar to the ventricular outflow tract structures, so it may act as the virtual teacher.

Notably, we just introduced how to use the model. The concrete correspondence between our model and the real heart needs to be explored by trainees. It also makes learning occur in an active process as constructivism requires.

### Study Goals

(1) This study aimed to explore whether our hands can create a model depicting the ventricular outflow tract for educational purposes. (2) We wondered about the experiences of the trainees using the two-handed model. (3) We hypothesized that this specific two-handed model may help electrophysiology trainees improve their understanding of ventricular outflow tract anatomy, contiguous structures, and associated electrophysiological knowledge. We advocate a pragmatic paradigm in this study. Pragmatism argues that problems are best defined by people who have experienced them, resulting in actionable research and that knowledge is related to that experience [26, 27]. In our study, trainees asked us for help learning ventricular outflow tract anatomy, leading to our actionable research. In turn, our experiences helped us propose alternative solutions. Pragmatism is the best way to investigate real‐world problems, and it allows the use of multiple data points in conducting the research [28]. Our study also included mixed methods and data. We collected quantitative data (test) corresponding to goal (1) and qualitative materials (questionnaire) corresponding to goal (2).

## Methods

The study protocol was reviewed and approved by the Health Sciences Research Ethics Board of the Office of Research Ethics, Chang Zhou Geriatric Hospital, Soochow University. All cardiologists who agreed to participate in the latest electrophysiological training course conducted at the First Affiliated Hospital of Nanjing Medical University and the Changzhou Geriatric Hospital affiliated with Soochow University were invited to participate in this study. The First Affiliated Hospital of Nanjing Medical University has one of the largest electrophysiological centers in China. The center has several well-known electrophysiologists and educators who have conducted many important studies [29–31] and held a national electrophysiology training course for more than 10 years. The main incentive for participation was the benefit of learning ventricular outflow anatomy. No monetary incentive was given.

The inclusion criteria for the study required participants to be working in the Division of Cardiology and to know little about cardiac electrophysiology. Experienced electrophysiologists were excluded from the study. All participants had been accepted into a five-year (10 semesters) medical program at the undergraduate level. They received comprehensive dissection teaching (systematic and topographic anatomy) during the first four semesters, but less was taught about electrophysiology-relevant anatomy, especially the outflow tract. The courses during the first four semesters comprised lectures, human and animal dissections, and anatomical model study. After students graduated from the undergraduate course of study, they received very little systematic anatomy teaching; rather, they mainly participate in clinical self-study. Trainees used to be mainly responsible for the drug treatment of cardiovascular diseases. Participants were randomly distributed into variable and control groups. Prior to the initiation of the study, knowledge of the ventricular outflow tract anatomy and associated electrophysiology of all participants was assessed. Teachers were blinded to group allocation and had no access to the collected data.

Participants in both groups underwent a one-week training course that included outflow tract anatomy theory, heart models, three-dimensional anatomical pictures, porcine heart anatomy, related information on electrophysiology, and a study of the literature. The whole course consists of 20 classes for a total of 20 h. The detailed course schedule can be found in Supplement 1. The week after the course, all trainees observed and participated in surgery. Before the one-week course started, the variable group was also introduced to the two-handed model. This additional instruction lasted only 5 min. We just told the trainees how to use the model by showing the most basic anatomical structures and encouraged them to find the one-to-one correspondence between the model and detailed structure in the following learning process. By contrast, the control group was taught in advance how to use the traditional model, which uses double upper limb forearm overlapping to clarify the anterior–posterior relationship of right and left outflow tracts. Limb model teaching lasts approximately 3 min. All participants were tested one day after the course and again one week later (participants were not informed of either test in advance). Our study was performed by conducting pre- and post‑tests on medical students because these tests can effectively assess the educational effect and learning gain [21, 32]. We scheduled a third unanticipated test to check the students' abilities to remember facts and terminology when needed ("retention") [33, 34]. We made use of the previous test (a total of 30 questions) in our training center, which was created many years before our idea. We designed this method to avoid incurring implicit bias. We originally planned to design a total of 60 questions (20*3) for the three tests. The quiz was designed to include all the key points of electrophysiology-related ventricular outflow tract anatomy and to be comprehensive rather than based on whether our model can be applied to the questions. We originally planned to design a total of 60 questions (20*3) for the three tests. After consulting many studies[1–7, 9, 35], however, only 10 questions were added for a total of 40 questions. As a result, the questions included in the first and second tests were identical. The third test included different questions that were almost as difficult as those on the first two tests. The comprehensiveness, validity, and level of difficulty of all three tests were empirically assessed by the well-known electrophysiologist and educator Minglong Chen. Each test consisted of 20 questions, including single, multiple-choice questions and fill-in-the-blank questions. The total score of each test was 100 points and covered the following sections: 1. The general spatial position of the heart (e.g., Is the heart in the thorax lying flat, oblique, or hanging?); 2. the interrelationship of the various components of the heart (e.g., Relative to the aortic root, the position of the pulmonary root is___?); 3. definition of special structures (e.g., AMC refers to the area bordered by __?); 4. anatomy-related electrophysiological basis (e.g., In the right coronary aortic sinus, which kind of wave can be recorded?); and 5. anatomically relevant electrophysiological clinical application (e.g., The anterior interventricular artery may be damaged during ablation at the __ part of right ventricular outflow tract?). Details of the test questions can be found in Supplement 2. Finally, we randomly surveyed 10 students and listed their views on the advantages and disadvantages of our model. Their input was integrated into a questionnaire (Supplement 3) that was administered to all trainees in the variable group. The questionnaire contained 6 advantages, 3 disadvantages and a general comment, with each scored 1–5 points (1. totally disagree 2. disagree a little 3. neutral opinion 4. agree a little 5. totally agree).

### Use of the two-handed model

**Fig. 1:**
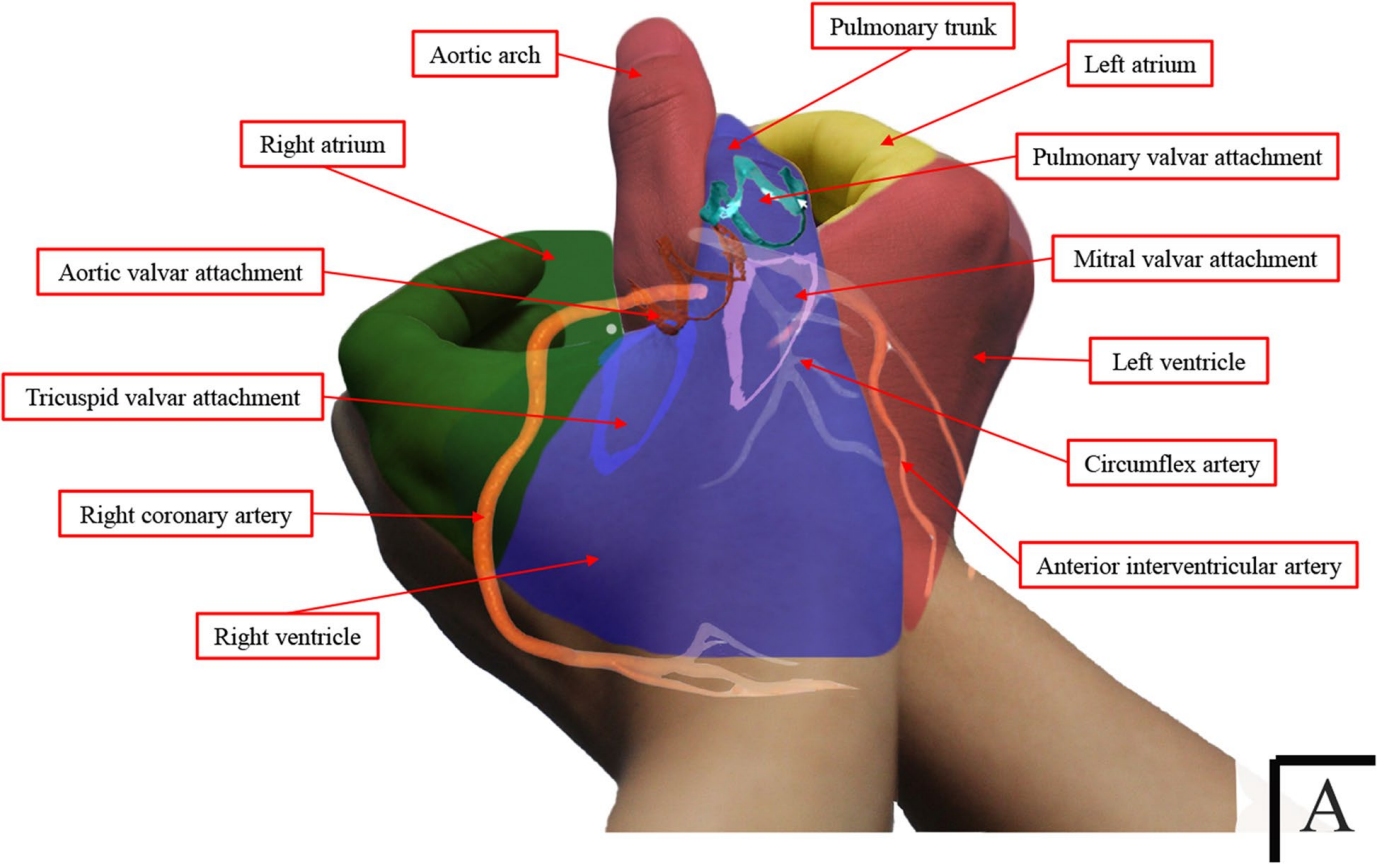
Representation of the two-handed model and one-to-one correspondence between the structures of the hand and the heart. The cardiac chambers have been segmented separately and colored while being placed in attitudinally appropriate locations. This image shows the relationships between the various components of the heart. A in the lower right corner refers to the anterior–posterior view

**Fig. 2:**
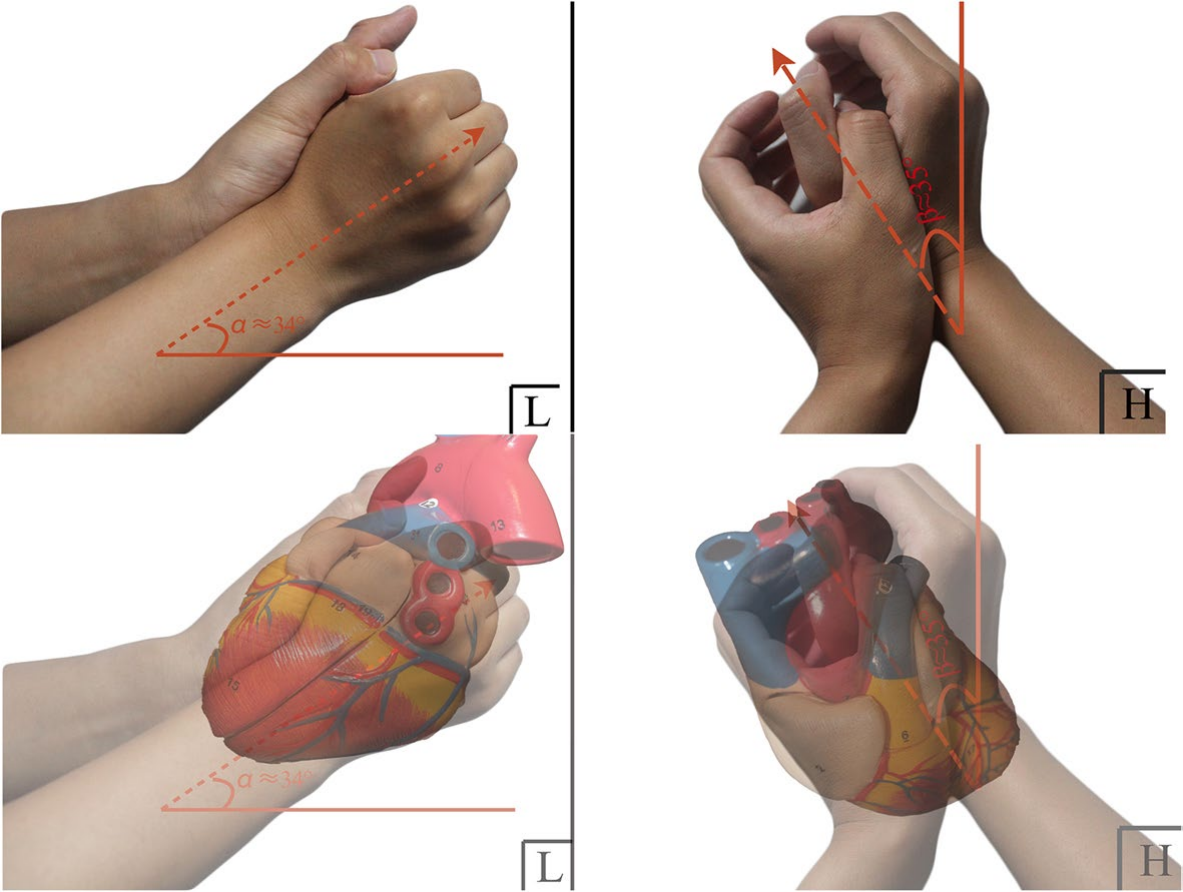
Images show how to achieve the two-handed model and indicate the specific angle in the lateral (left panel) and horizontal (right panel) planes of the thorax. Two pictures below include an overlay of the heart model to reinforce these two planar views. L/H in the lower right corner refers to the left-lateral/head view

To visualize the two-handed model, imagine playing a hand shadow game and creating a bird that is spreading its wings to fly by making a "C" shape with the left hand, its mirror image with the right hand, and linking the thumbs together. By rotating the hands back and forth with the bird’s abdomen (palm of both hands) back against yourself, a shape similar to that of the ventricular outflow tract is achieved (Fig. 1). Additionally, the hands, forearms, and all their joints need to be fine-tuned to a certain angle (Fig. 2). Most importantly, the model should be designed in an attitudinally appropriate fashion. A one-to-one correspondence between the following structures of the hand and the heart is observed in Fig. 1: the left/right palms versus the right ventricle (RV)/left ventricle (LV); the left thumb-index finger webspace versus the atrioventricular groove or the attachment of the anterior leaflet of the tricuspid valve; the space from the left/right index finger to the little finger versus the right/left atrium; the junction of the left first metacarpophalangeal joint and the first web space versus the membranous part of the interventricular septum or the penetrating bundle of His; the left first metacarpophalangeal joint versus the supraventricular crest; the proximal phalanx of left thumb versus the freestanding pulmonary infundibulum; the distal phalanx of left thumb versus the pulmonary trunk; the interphalangeal joints of the left thumb versus the pulmonary root containing pulmonary sinuses and leaflets; the right first metacarpophalangeal joint versus the aortic root containing aortic sinuses and leaflets; the proximal phalanx of the right thumb versus the ascending aorta; the distal phalanx of the thumb versus the aortic arch; and the lateral margin of the left thenar eminence where both hands overlap versus the anterior interventricular sulcus.

**Fig. 3:**
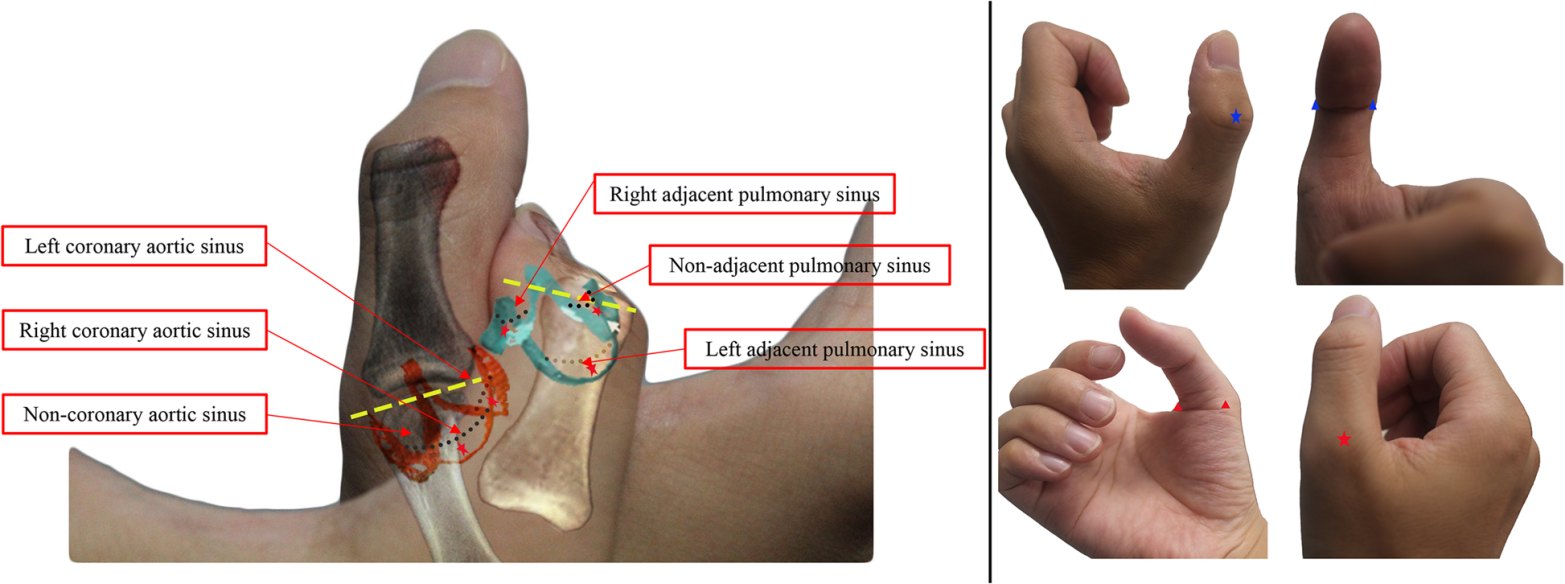
A "blown up" image (left panel) of the aortic root and the pulmonary artery root in the two-handed model. The dashed yellow line marks the direction of the joints, which is consistent with the direction of the aortic/pulmonary sinus. According to this characteristic, we can infer that the noncoronary aortic sinus is the most inferior; the left coronary aortic sinus is the most superior; and the left adjacent pulmonary sinus is inferior to the other two pulmonary sinuses. The right panel shows native bony protrusions (stars), and intersection angles (triangles) can be regarded as three aortic/pulmonary sinuses. A one-to-one correspondence: blue star versus nonadjacent/anterior pulmonary sinus; left/right blue triangle versus left/right pulmonary sinus; red star versus right coronary sinus; left/right red triangle versus left/noncoronary sinus. However, the model cannot depict cardiac histology. For example, the dashed white lines form the sinutubular junction. The dotted black line shows the ventriculo-arterial junction. The area beneath the dotted black line (red stars) shows the myocardial crescents incorporated at the bases of the aortic/pulmonary sinuses

The following anatomical characteristics and electrophysiological information can be deduced from the two-handed model: 1. The long axis of the ventricle is directed from right posterior to left anterior, with an additional inferior tilt. 2. The apex of the heart is composed of the LV and RV, of which the RV is dominant. 3. The RV lies anterior to the LV, while the left atrium is located at the posterior-most aspect of the heart. 4. The right ventricular outflow tract is anterior and leftward relative to the left ventricular outflow tract. 5. The LV outlet is more abbreviated than that of the RV. 6. The pulmonary root is placed superior to the aortic root. 7. The most inferior pulmonary valvar sinus is the left adjacent pulmonary sinus, deduced from the oblique direction of the interphalangeal joint of the left thumb (Fig. 3). 8. The left coronary aortic sinus is superior to both the right coronary aortic sinus and the noncoronary aortic sinus, as shown by the direction of the right first metacarpophalangeal joint (Fig. 3). 9. The left anterior descending artery/anterior interventricular vein runs along the lateral edge of the left thenar eminence. We believe that the anterior interventricular vein is a tributary or continuation of the great cardiac vein actually coursing within the anterior interventricular groove. The right coronary artery is located at the left thumb-index finger web space. The right coronary aortic sinus faces the RV. The anterior pulmonary sinus (nonadjacent pulmonary sinus) is located at the anterior-most aspect of the three pulmonary valvar sinuses, and the left adjacent pulmonary sinus is adjacent to the left coronary aortic sinus. By integrating the above information, it is possible to determine the relative positions of the three aortic sinuses. During a radiofrequency catheter ablation procedure, the two-handed model can be rotated such that right anterior oblique (RAO) 30°, left anterior oblique (LAO) 45°, left lateral (LL), and posteroanterior (PA) views are reflected, which helps practitioners understand the position of each aortic sinus and pulmonary sinus at all angles. 10. The aortic sinus is close to the bundle of His, which is illustrated in the model by the adjacent positions of the first metacarpophalangeal joints of the left and right hands. 11. The anterior interventricular artery and the anterior interventricular vein run at the posterior aspect of the right ventricular outflow tract. 12. Intracardiac electrogram characteristics of the aortic sinus can be inferred from the tissues adjacent to each sinus of the aorta. For instance, large A waves can be recorded in the noncoronary aortic sinus. 13. Analyzing the site of origin of ventricular arrhythmias can aid in predicting electrocardiogram characteristics. 14. Other special anatomical structures, such as the aortomitral continuity, the LV summit, and the aortic-mitral curtain, can be visualized. After memorizing the features depicted in Fig. 1, only basic knowledge of anatomical features, such as the components of the aortic sinuses (including the noncoronary aortic sinus, left coronary aortic sinus, right coronary aortic sinus), the components of the pulmonary valvar sinuses (including the anterior, left adjacent, and right adjacent sinus), and the LMCA arising from the left coronary aortic sinus, is needed. The above knowledge was summarized by the authors (XX, YJ) after experiencing the two-handed model. We took the most basic structures as an example to introduce our model to the trainees. Trainees need to explore the model themselves during the learning process.

### Data collection and analysis

Three authors (XX, XYL, YJ) checked the answer sheets and calculated the scores. For fill-in-the-blank questions, there may be different criteria for correct answers. All authors discussed whether such answers were correct. The results were discussed until a consensus was achieved. All authors proposed various opinions and ensured increased credibility. As a third-party investigator, Dr. Yang reviewed and examined the research process and data analysis to ensure that the findings were consistent and could be repeated.

### Statistical analysis

The sample size was calculated using PASS 15.0. Group sample sizes of 57 and 57 achieve 80% power to reject the null hypothesis of equal means when the participants’ mean difference is μ1—μ2 = 75.3–82.1 = -6.8 with standard deviations of 14.1 for Group 1 and 11.2 for Group 2 and with a significance level (alpha) of 0.050 using a two-sided, two-sample, unequal-variance t test. We determined that the sample size of 19 trainees in each group (a total of 38 trainees) would give the trial a power of 36%. We will address this point in the limitations. Data were analyzed using SPSS version 26.0 for Windows (IBM Corp., Armonk, NY). Continuous variables are presented as the mean ± SD or median (P_25_, P_75_), as appropriate. Exam data are ranked data, thus the nonparametric Mann–Whitney U test was used to assess the difference between the two groups and Hodges–Lehmann was used to estimate of confidence intervals for pseudo-medians. The Wilcoxon signed-rank test was used to assess the difference between different times in the same group. Statistical significance was set at *p* < 0.05.

## Results

A total of 38 trainees were included in the study, with 19 trainees each in the control and variable groups. The average age of the enrolled trainees was 35.4 ± 5.1 years old; there were 18 males, accounting for 47.4% of the trainees; the average number of working years was 10.4 ± 5.5 years; and 27 people had a master’s degree or above, accounting for 71.1% of the trainees. No significant between-group differences in academic degree, age, or sex were observed (Table 1). All participants completed the study between February 14 and February 28, 2022.

**Table 1:** Between-group comparison of trainee characteristics at baseline.

Characteristic	Control (*n *= 19)	Variable (*n* = 19)	*p value*
Age, mean ± SD (years)	35.1 ± 5.3	35.7 ± 5.1	0.710
Male sex, n (%)	6 (31.6)	12 (63.2)	0.051
Work experience, median (P_25,_ P_75_) (years)	10 (7,11)	10 (7,12)	0.583
Master’s degree and above, n (%)	14 (73.7)	13 (68.4)	0.721

Trainees received a median score of 38 (24,55) points on the test administered prior to the course initiation (control, 45 (25,50) points; variable, 30 (20,55) points). One day and one week after course completion, the median score among all study participants was 80 (70,86) points (control, 70 (65,85) points; variable, 80 (75,95) points) and 75 (70,81) points (control, 70 (65,75) points; variable, 75 (75,90) points), respectively.

**Fig. 4:**
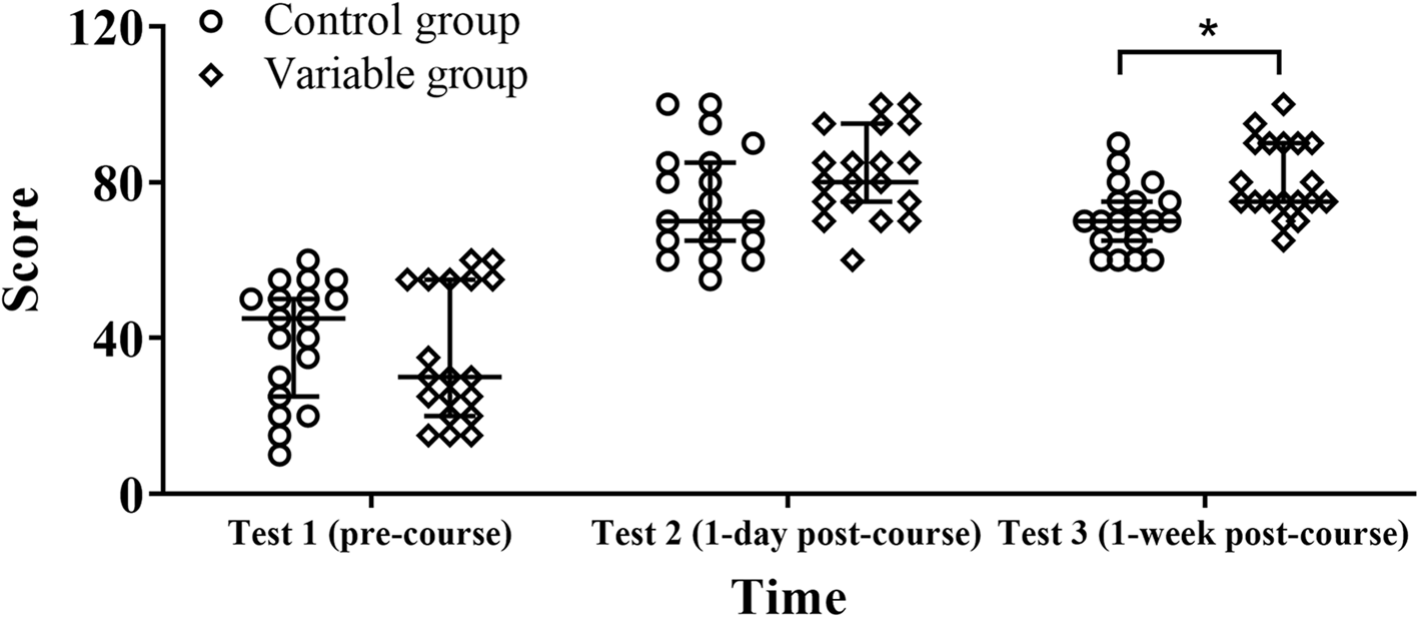
Comparing the median scores of tests 1–3 between the two groups. Trainees in both groups were tested 3 times (pre-course, 1 day post-course, 1 week post-course). The test scores of each participant are marked in the figure with a circle or square. The three horizontal lines of each group correspond to P_25_, median, and P_75_ (bottom to top). _*_ indicates that the difference is significant at the 0.05 level

No significant between-group differences in the pre-course test scores were observed (z = -0.412, *p* = 0.686). For both groups, test scores achieved one day after course completion were significantly better than those achieved prior to the course (control, z = -3.730, *p* < 0.001; variable z = -3.835, *p* < 0.001). Furthermore, trainees who received instruction using the two-handed model scored a mean 6.8 points higher than those who did not, a difference that was not significant (z = 1.662, *p* = 0.103; Table 2). One week after course completion, the scores of both groups marginally declined; however, the decrease was only significant in the control group (control: z = -2.159, *p* = 0.031; variable: z = -1.032, *p* = 0.302). Notably, trainees in the variable group had a mean score 9.7 points higher than those in the control group one week post-course completion (z = 2.955, *p* = 0.003; Table 3). The findings are summarized in Fig. 4.

**Table 2:** Comparison of pre-course and one day post-course test scores.

	Control (*n* = 19)	Variable (*n* = 19)	Difference (95% CI)	*z*	*p value* (control versus variable group scores)
pre-course score	45(25, 50)	30(20, 55)	0 (-5,20)	- 0.412	0.686
1 day post-course score	70(65, 85)	80(75, 95)	-10 (-15, 0)	1.662	0.103
Difference (95% CI)	-35 (-45, -25)	-45 (-60, -35)			
*z*	-3.730	-3.835			
*p value* (pre- versus post-course score)	< 0.001	< 0.001

**Table 3:** Comparison of test scores at one day and one week post-course completion.

	Control (*n* = 19)	Variable (*n* = 19)	Difference (95% CI)	*z*	*p value*(control versus variable group scores)
1 day post-course score	70(65,85)	80(75,95)	-10 (-15,0)	-1.662	0.103
1 week post-course score	70(65,75)	75(75,90)	-10 (-15, -5)	2.955	0.003
Difference (95% CI)	5 (-5,10)	0 (-5,10)			
*z*	-2.159	-1.032			
*p value*(1 day versus 1 week post-course score)	0.031	0.302

**Fig. 5:**
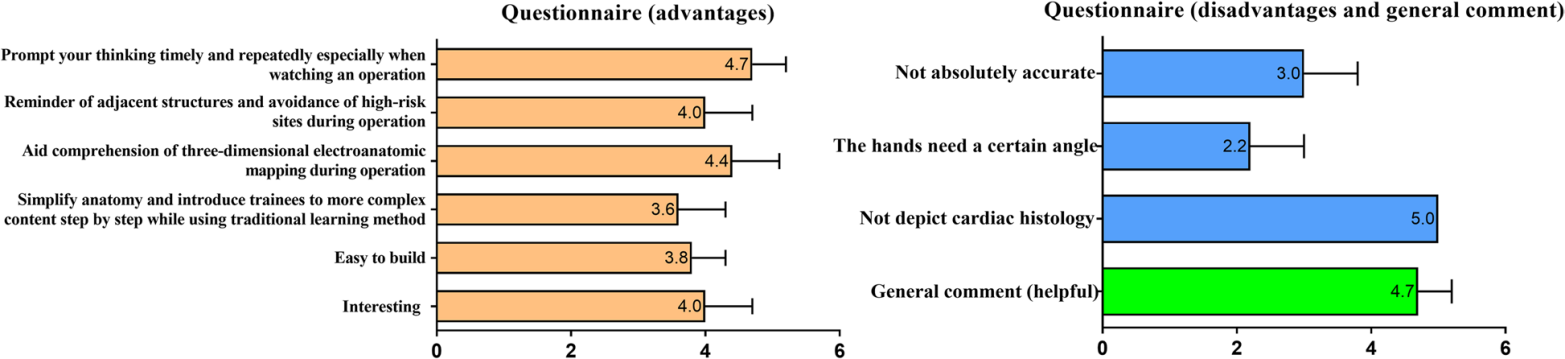
Questionnaire of the trainees’ views on the advantages and disadvantages of our model. A questionnaire containing 10 items was administered to all trainees in the variable group. The items contained 6 advantages (left panel), 3 disadvantages and a general comment (right panel). Each item was scored 1–5 points (1. totally disagree, 2. disagree a little, 3. neutral opinion, 4. agree a little, and 5. totally agree). The numbers on the right of each horizontal bar represent the mean score for each item. The graph shows standard deviation error bars

In the advantages section of the questionnaire, the items *Prompt you to think timely and repeatedly, especially when watching an operation*; *Aid comprehension of three-dimensional electroanatomic mapping during operation*; and *Reminder of adjacent structures and avoidance of high-risk sites during operation together* with *Interesting* were ranked as the top three advantages (4.7 ± 0.5, 4.4 ± 0.7, 4.0 ± 0.7, 4.0 ± 0.7 points, respectively). In the disadvantages section of the questionnaire, the item *Not depict cardiac histology* ranked first as the top disadvantage (5.0 ± 0.0 points). The general comment (helpful) scored 4.7 ± 0.5 points. Details of the results can be found in Fig. 5.

## Discussion

It is often difficult for trainees to become proficient in ventricular outflow tract anatomy. Although all trainees included in this study had majored in anatomy in medical school and had worked in a clinic for several years, the test scores achieved prior to the course were very low. Traditional anatomy instruction combined with the two-handed model improved the test scores of trainees, especially those obtained one week after course completion.

Knowledge of anatomy is important for medical practitioners. Anatomy textbooks and cardiac models are the most commonly used teaching modalities. Cadaveric dissection as an alternative method is difficult to perform in many electrophysiology study centers due to limited availability, while porcine heart dissection is an intuitive and accessible alternative. However, the shape and components of a pig heart differ from those of a human heart [36]. Virtual reality, body painting and 3D visualization approaches are relatively novel teaching methods. Some reports demonstrate their effectiveness [37, 38]. Virtual dissection of three-dimensional datasets obtained via computed tomography is particularly recommended due to its accuracy and appropriateness [39]. New teaching methods are unquestionably welcomed because they have the potential to increase trainees’ interest and boost initiative [40].

However, the abovementioned new approaches are not common in hospitals. There are several reasons: 1. These approaches may lead to negative effects. Favorable attitudes or levels of engagement do not necessarily correlate with enhanced and sustained learning outcomes [41]. 2. Interesting methods do not necessarily correspond to more learning time. They may distract students from traditional learning. The effects are sometimes determined by individual differences among students [42]. 3. Theoretical teaching in hospitals accounts for a smaller proportion than in medical schools. Knowledge about anatomy is often integrated and applied in different clinical courses. The cost-effectiveness is low. 4. The flaw in most of the above methods is that they are challenging to use during ablation. Therefore, traditional teaching methods still dominate in most hospitals, including our electrophysiology center. In contrast, the two-handed model is cost-free. It is designed in an attitudinally appropriate fashion and can be rotated, split, and used anywhere. It may increase study time by prompting students to think repeatedly. Therefore, the two-handed model is an effective alternative that can help students obtain knowledge of cardiac outflow tract anatomy.

Poor performance on initial tests indicates that greater expertise in outflow tract anatomy, contiguous structures, and electrophysiology is needed. An analysis of the specific questions revealed that some questions regarding basic knowledge of the cardiac anatomy were often answered correctly; however, only a few questions regarding knowledge of anatomical details and adjacent structures were answered correctly. This indicated that cardiologists require specific and intensive training prior to engaging in electrophysiological work. In this study, after systematic instructions, the scores of both groups improved significantly, with the scores of the variable group marginally (and not significantly) higher than those of the control group. This indicates that traditional learning methods (Supplement 1), such as anatomical theory combined with heart-model-based instruction, porcine heart dissection, and a study of the literature, can be highly effective.

As the questions of the first and second tests were identical, trainees might have been alerted to particular subject areas during the course, which may have contributed to a lack of significant difference observed between the mean second test scores of the two groups. Notably, one week after the course, test score reduction was more obvious in the control group, whose members underwent traditional training exclusively. In contrast, trainees who received combined traditional and two-handed model training continued to perform at a high level. One week after course completion, the mean test scores of trainees who used the two-handed model was significantly better than those of the control group who did not use the model, demonstrating that the conventional teaching method combined with a two-hand model promotes knowledge retention.

We speculated on several reasons why the two-handed model may promote knowledge retention. 1. Some doctors did not effectively consolidate their memories after the course. Therefore, after one week, a period inspired by the Ebbinghaus forgetting curve [43], scores declined. Although the first, second and third tests were equally difficult, differences in questions required students to rationally consider their answers. 2. Learning the outflow tract anatomy is rather tedious. According to the author’s experience and trainee feedback, the hand model improved the learning experience in a manner similar to that of body painting, by increasing the visual and sensory stimuli [44]. 3. When a patient with relevant anatomy is encountered either intra- or postoperatively, knowledge validation via the two-handed model is possible, which facilitates knowledge consolidation. While trainees who did not learn the two-handed model intended to do additional research at a later time, they often opted to perform other tasks. 4. Although some trainees in the variable group had forgotten some key points by the time they took the third quiz, the two-handed model helped them recall specific facts.

According to the questionnaire, trainees believed that the model has several advantages. In general, the model is interesting and easy to build; it simplifies the outflow tract anatomy and introduces students to more complex content step by step; it is not limited by time or place, thus prompting students to think repeatedly, especially when watching an operation; and it can be used during ablation without violating the aseptic principle. This feedback is consistent with our theoretical basis. Constructivism calls for exploration, coaction, experimentation, construction, and reflexion in the study process [21]. After learning our model, trainees tried to find the one-to-one correspondence between the model and structure. This interaction that ran through all learning processes was a little like doing a crossword puzzle. Trainees explored the model, constructed the correspondence, communicated with editors or friends about the model, and thought about the anatomy repeatedly during the ablation the next week. As a result, they were more proactive in learning and might have increased their total time of study. In addition, trainees were supported by the model liked "scaffolding" and became more confident [45]. Hence, trainees were able to overcome the difficulties at the beginning and learn more complex content step by step.

Finally, our model still has room for optimization. A teacher said that adding body painting to the hands with a label shown in Fig. 1 might have a greater impact. We initially did not include this because the model's greatest feature was that it could be used anywhere and anytime, especially intraoperatively. Painting would violate the aseptic principles of the operating room. Another reason was based on our original intention to create this model. As we have stated in the methods, the instruction of how to use this model, which only lasted approximately 5 min, was straightforward. We taught the students how to use it by taking the most basic structures as an example. We encouraged students to find the one-to-one correspondence between the hand model and the detail structures during the subsequent learning process. Thus, we did not show the detailed structures directly by painting and labeling the hands. However, painting our model may be particularly suited to anatomy teaching in medical schools. We are eager to demonstrate the use of a hand model with painting for educational purposes in further study.

### Limitations

Although this study is informative, it has some limitations. First, as Anderson et al. stated, some teaching modalities cannot generate an anatomically accurate heart [46], and we have similar concerns about our model. Although our proposed model only requires two hands for its creation, its accuracy cannot be compared to anatomy textbooks and clinical imaging, and the model is not error-free. This is also reflected in the scores of the second test between the two groups after a standardized one-week teaching period, wherein no difference was reported. We believe that the model is not designed to replace any existing method but is only an important supplement. The premise of using the model is to recognize its shortcomings. Second, the model is merely a morphological representation and does not depict the cardiac developmental process and histology (Fig. 3). The latter is extremely important for electrophysiologists, as the objective of learning anatomy is to understand the mechanisms of underlying arrhythmias and to guide treatment. For instance, correct localization of the myocardial components in the ventricular outflow tract will aid in the intervention of arrhythmias [47]. On analyzing the reconstructed clinical imaging and heart specimens, the crescents of the myocardium in the aortic root are found only at the bottom of the right coronary aortic sinus and the anterior half of the left coronary aortic sinus. In contrast, crescents are found at the base of each pulmonary valvar sinus [48]. Together with the fibro-adipose tissue plane extending from the left ventricular free wall to the crest of the muscular ventricular septum, the myocardium supporting all three leaflets of the pulmonary valve forms the freestanding pulmonary infundibular sleeve [1]. It is the infundibular sleeve that lifts the pulmonary artery root away from the base of the LV [49]. The abovementioned foci are potential arrhythmic substrates and are extremely important to electrophysiologists. In addition, myocardial tissue lying on the epicardial aspect of the valvar sinuses, anatomic ventriculo-arterial junction, and various other locations also need attention. Unfortunately, their localization is not possible in the proposed two-handed model, so the latter must be combined with other learning methods. Third, tests one and two had the same set of questions, which may have prevented the appearance of significant between-group differences. Fourth, relatively few trainees participated in the study; thus, there was not sufficient power or validity. As pragmatists, we think this topic may be of interest because it comes from the desire of trainees and can solve practical problems in the real world [50]. All of our authors were electrophysiological trainees in the previous round and had experienced the model. We proposed reasonable assumptions by experiencing the resulting outcomes [51]. As our experience only takes place within particular contexts [52], we designed this research to demonstrate the use of the hand model specifically for electrophysiological trainees. The First Affiliated Hospital of Nanjing Medical University owns the largest electrophysiological center in China. It recruits a group of new trainees every year, and the number fluctuates between 35 and 45. To meet the preconditions of being a new electrophysiology trainee and accepting the same curriculum, the current number is the upper limit we can include. As a new method has never been proposed, it is challenging to conduct research in multiple centers with a larger sample, and we are afraid that the education received is inconsistent. When more doctors are aware of our study, additional studies with larger sample sizes will be needed for validation. Finally, this study only examined the model’s suitability for trainees who are beginners in electrophysiology. The usefulness of the two-handed model to cardiologists with experience in radiofrequency catheter ablation and undergraduate students in medical school is yet to be determined. However, we must make it clear that the limitations do not devalue the two-handed model as a virtual teacher of anatomy. We proposed the above limitations to remind users that they must understand more than the morphology of cardiac anatomy. The model is mainly expected to help students transition to more challenging content, which in turn makes up for the limitations of the model. Therefore, the two-handed model and other teaching methods are complementary. The above expectations have little to do with the limitations.

## Conclusions

In summary, this study demonstrates the use of hands to create a model depicting the ventricular outflow tract for educational purposes. The model has the advantage of easy establishment and use at any time and place. Our findings indicate that this model improves comprehension of the ventricular outflow tract anatomy and reinforces knowledge retention because it introduces students to more complex content step by step and prompts students to think repeatedly. For trainees beginning to study electrophysiology, educators could tell them how to use the two-handed model as an important supplement to traditional teaching methods. Trainees should incorporate the model into the whole process of heart anatomy learning. However, the limitations of the two-handed model need to be addressed in advance. Whether this model is suitable for anatomy teaching for medical students and experienced electrophysiologists still needs further research.

## Supplementary Information

**Additional file 1:** Course schedule details.

**Additional file 2:** Questions for each test.

**Additional file 3:** Questionnaire.

## Data Availability

The dataset used during the current study is available from the corresponding author upon reasonable request.
