# Supplementary material for: Use of a two-handed model to improve comprehension of ventricular outflow tract anatomy
Source: BMC Med Educ. 2023 Feb 8;23:101. doi: 10.1186/s12909-023-04083-w (PMC9909947; doi:10.1186/s12909-023-04083-w)
Supplement: Supplementary file 1 — Additional file 1: Course schedule details. [file 12909_2023_4083_MOESM1_ESM.docx]

**Course schedule details**

**Day1**

Content: General cardiac anatomy

Duration: 4 classes, 1 hour/class

Teaching material:

1)Wenlong Ding, Xuezheng Liu.(2018)Systematic Anatomy. Beijing: People's Medical Publishing House.

Note：Through AR technology, the plan in this textbook is stereoscopic, and the abstract knowledge is transformed into an easy to observe 3D learning scene.


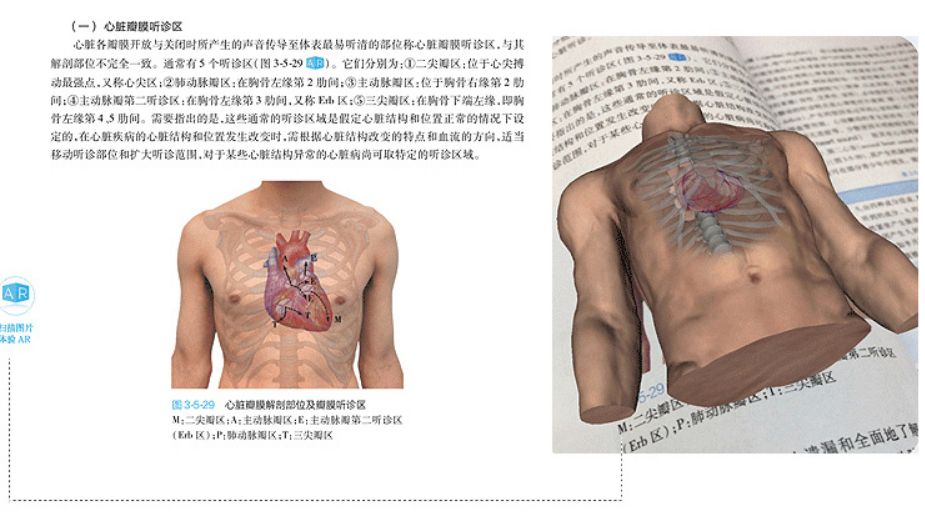


2)PVC Heart model


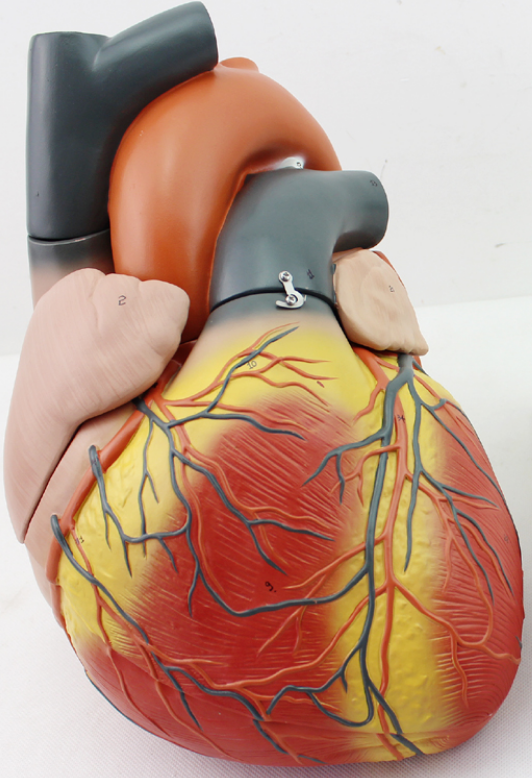


**Day2**

Content: literature study of General cardiac anatomy

Duration: 4 classes

Teaching material:

1)Mori S, Tretter JT, Spicer DE, Bolender DL, Anderson RH. What is the real cardiac anatomy? Clin Anat. 2019 Apr;32(3):288-309. Doi: 10.1002/ca.23340.

2) Mori, S. and K. Shivkumar(2021). "Stereoscopic three-dimensional anatomy of the heart: another legacy of Dr. Wallace A. McAlpine." Anat Sci Int 96(3): 485-488.

**Day3**

Content: Three-dimensional anatomical pictures

Duration: 2 classes

Teaching material: Anatomical 3D atlas and resource from the Internet

**Day4**

Content: Rest and review general cardiac anatomy

**Day5**

Content: The anatomy of the ventricular outflow tract

Duration：4 classes

Teaching material:

1)Asirvatham SJ. Correlative anatomy for the invasive electrophysiologist: Outflow tract and supravalvar arrhythmia. J Cardiovasc Electrophysiol 2010; 20:955-968. DOI: 10.1111/j.1540-8167.2009.01472.x

2)Chun KR, Satomi K, Kuck KH, Ouyang F, Antz M. Left ventricular outﬂow tract tachycardia including ventricular tachycardia from the aortic cusps and epicardial ventricular tachycardia. Herz 2007;32:226-232. DOI: 10.1007/s00059-007-2977-0

3) Li YG, Gronefeld G, Israel C, Hohnloser SH. Sustained monomorphic ventricular tachycardia ablation from the aortic sinus of valsalva. J Cardiovasc Electrophysiol 2002;13:130-134. DOI: 10.1046/j.1540-8167.2002.00130.x

4) Sidhu S, Calkins H. New insights on ablation of idiopathic ventricular arrhythmias arising from the right ventricular outflow tract. Heart Rhythm 2019;16:1521-1522. DOI: 10.1016/j.hrthm.2019.04.046

5) Katie AW, Gerard JF. Anatomy of the left main coronary artery of particular relevance to ablation of left atrial and outflow tract arrhythmias. Heart Rhythm 2014;11:2231-2238. DOI: 10.1016/j.hrthm.2014.08.006.

Note: We have integrated the knowledge points and related graphs in the above literature into our teaching plan.

**Day6**

Content: Porcine heart anatomy

Duration：2 classes

Teaching material: Porcine heart

Note: The goal is that every trainee can find all structures by themselves and

master adjacent relationships.

**Day7**

Content: Ventricular outflow tract anatomy and related electrophysiological knowledge

Duration：4 classes

Teaching material: Book:S. Yen Ho. Anatomy For Cardiac Electrophysiologists，Published August 2012,ISBN: 9780979016448


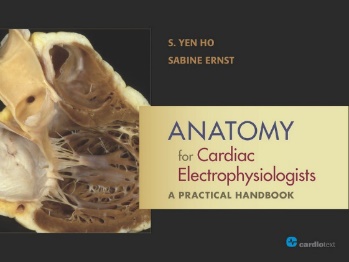


Note: We focused on the relationship between anatomy and intracardiac electrogram and its correlation with ablation.
