# Supplementary material for: Use of a two-handed model to improve comprehension of ventricular outflow tract anatomy
Source: BMC Med Educ. 2023 Feb 8;23:101. doi: 10.1186/s12909-023-04083-w (PMC9909947; doi:10.1186/s12909-023-04083-w)
Supplement: Supplementary file 2 — Additional file 2: Questions for each test. [file 12909_2023_4083_MOESM2_ESM.docx]

**Test for the first and second time**

1.Which option is correct about the apex of the heart ()?

A face towards anterior left inferior B formed by the RV and LV

C is predominantly composed of the RV D is predominantly composed of the LV

2. Which part of the RVOT is adjacent to the aortic root?

A right front part B free wall

C left part D posterior part

3. Which aortic sinus is adjacent to the proximal RVOT (posterior part)?

A RCC B LCC C NCC

4. The septal part of the RVOT refers to ()?

A right front part B free wall

C left part D posterior part

5. Is the septum of RVOT part of the ventricular septum?

A yes B no C uncertain

6.The most common intracardiac electrogram recorded in the NCC is

A aV B Av C av

7.In the coronal Image reconstruction of heart, the anatomical structure of the central position is

A proximal RVOT B aortic root C pulmonary valve D right atrium

8.Where is the location of his bundle?

A between NCC and RCC B between NCC and LCC

C between RCC and LCC D no correlation with AS

9.The His bundle is presumably positioned in the () o'clock direction at the tricuspid annulus

10.In the RAO 30° view，the anterior two aortic sinuses from left to right are (), () (refer to the left and right of the trainees)

11.The three sinuses of the aortic sinus are (), (), ().

12. The three valves of pulmonary valve are (), (), ().

13. The most posterior aspect of the heart cavity is (), and the most anterior cavity is ()?

14. The anatomical definition of the dividing line between the inflow tract and the outflow tract of the right ventricle is ().

15. The systemic circulation starts in the () and ends in the (); The pulmonary circulation begins in the () and ends in the (); The heart's own blood supply starts in the () and ends in the heart ().

16.RVOT is () and () to the LVOT. (Responses on anterior posterior and left-right relations, respectively)

17.The three valves of the tricuspid valve are (), (), () . The His bundle is found between the (), ().

18.In the AP projection, the most anterior pulmonary valve is (), and the two posterior pulmonary valves are (), () from left to right (referring to the trainee's left and right).

19.Please describe the change of QRS complex in lead V1 when the origin of PVC varies from the anterior portion to the posterior portion.

20.Is the posture of the heart in the thorax lying flat, oblique, or hanging? What will happen in obese patients and in thin patients?

**Test for the last time**

1.In the RCC, which kind of wave can be recorded?

A aV B Av C av

2. In the LCC, which kind of wave can be recorded?

A aV B Av C av

3.compared with aortic valve, the position of the pulmonary valve is()?

A higher B lower C same

4. The location of the His bundle penetrating ​to the left ventricle is()?

5.In the LAO45° view, the two aortic sinuses from left to right are (), () (refer to the left and right of the trainee).

6.In the AP view, the most anterior aortic valve is (), and the two posterior aortic valves are (), () from left to right (referring to the trainee's left and right).

7. In the RAO30° view, the anterior two pulmonary valves from left to right are (), () (refer to the left and right of the trainee).

8. In the LAO45° view, the anterior two pulmonary valves from left to right are (), () (refer to the left and right of the trainee).

9.Among the three pulmonary valves, which one () is the lowest?

10. Among the three aortic sinuses, which one () is the highest?

11. Among the three aortic sinuses, which one () is the lowest?

12.Among the aortic sinus and pulmonary sinus, the two valves adjacent to each other are () and ()?

13. () can be considered as an ablation target for atrial arrhythmias originating in the His region?

14. () can be considered as an ablation target for ventricular arrhythmias originating in the His region?

15. LMCA may be damaged during ablation at the () part of RVOT.

16. LAD may be damaged during ablation at the () part of RVOT.

17. The PVC originating in GCV and LV summit can be considered to be ablated in the () part of the ROVT?

18.Tell the anatomical structure corresponding to 1 to 5 as outlined in Figure.


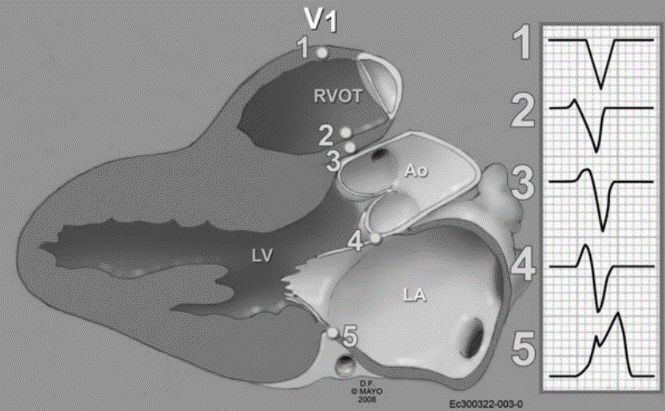


19. The proximal and mid segments of the RVOT are mostly located () to the aortic artery. The distal segment of the RVOT is mostly located () to the aorta.

20. AMC refers to the area bordered by the (),(),().
