# Supplementary material for: Use of a two-handed model to improve comprehension of ventricular outflow tract anatomy
Source: BMC Med Educ. 2023 Feb 8;23:101. doi: 10.1186/s12909-023-04083-w (PMC9909947; doi:10.1186/s12909-023-04083-w)
Supplement: Supplementary file 3 — Additional file 3: Questionnaire. [file 12909_2023_4083_MOESM3_ESM.docx]

**Questionnaire**

| **advantages** | **score** | **disadvantages** | **score** | **General** | **score** |
| --- | --- | --- | --- | --- | --- |
| Interesting |  | Not depict cardiac histology |  | Helpful |  |
| Easy to build |  | The hands need a certain angle |  |  |  |
| Simplify anatomy and introduce trainees to more complex content step by step while using traditional learning method |  | Not absolutely accurate |  |  |  |
| Aid comprehension of three-dimensional electroanatomic mapping during operation |  |  |  |  |  |
| Reminder of adjacent structures and avoidance of high-risk sites during operation |  |  |  |  |  |
| Prompt your thinking timely and repeatedly especially when watching an operation |  |  |  |  |  |

Each item scores 1-5 points (1. totally disagree 2. disagree a little 3. neutral opinion 4. agree a little 5. totally Agree).
